# Supplementary material for: Long-range versus short-range effects in cold molecular ion-neutral collisions
Source: Nat Commun. 2019 Nov 28;10:5429. doi: 10.1038/s41467-019-13218-x (PMC6882903; doi:10.1038/s41467-019-13218-x)
Supplement: Supplementary file 1 — Supplementary Information [file 41467_2019_13218_MOESM1_ESM.pdf]

Supplementary Information: Long-range versus short-range effects in cold molecular ion-neutral collisions

Alexander D. Dörfler[1], Pascal Eberle[1], Debasish Koner[1], Michał Tomza[2*], Markus Meuwly[1*] and Stefan Willitsch[1*]

[1] Department of Chemistry, University of Basel, Klingelbergstrasse 80, 4056 Basel, Switzerland
[2] Faculty of Physics, University of Warsaw,Pasteura 5, 02-093 Warsaw, Poland
∗ Electronic mail: michal.tomza@fuw.edu.pl, m.meuwly@unibas.ch, stefan.willitsch@unibas.ch

Supplementary Note 1 Determination of state-specific rate coefficients

Pseudo-first-order (pfo) rate coefficients $k_{x,\mathrm{pfo}}$ ($x = \mathrm{N}_2^+$ or O_2^+) for the CT reactions were obtained from monitoring the decrease of the number N_x of molecular ions over the time of reaction t and fitting the data to an integrated pseudo-first order rate law [1]:

$$\ln\left(\frac{N_x(t)}{N_x(t_0)}\right) = -k_{x,\mathrm{pfo}}t \tag{1}$$

This procedure was performed for all reaction measurements in all three operational modes of the experiment.

In the static operation mode of the MOT, the effective second-order rate coefficient dependent on the excited-state fraction discussed in the main text was calculated using

$$k = \frac{k_{x,\mathrm{pfo}}}{n_{\mathrm{avg}} f_{\mathrm{o}}} \tag{2}$$

where n_{avg} is the average density of the Rb atom cloud and f_{o} is a geometric overlap factor between the ions and atoms [2]. The Rb excited-state population p_p and n_{avg} was calculated as in Ref. [2] and the Rb ground-state population was obtained as $p_s = 1 - p_p$. State-specific rate coefficients k_s and k_p for reactions in the Rb $(5s)\ ^2S_{1/2}$ and $(5p)\ ^2P_{3/2}$ states, respectively, were obtained from measuring k for a range of different values for p_s and p_p and fitting the data to

$$k = \frac{1}{2}\left(k_s(1+p_s) + k_p p_p\right). \tag{3}$$

In the dynamic operation mode of the MOT, the rate coefficient was obtained using equation (2) for the experiments in which the transverse Rb cooling laser beams were switched off while the atoms transited the ions (dark shuttling mode). In this case, the Rb population was entirely confined to the ground state so that $k \equiv k_s$. Here, n_{avg} was determined from equation (2) in a reference measurement of $k_{\mathrm{O}_2^+,\mathrm{pfo}}$ using the shuttling atom cloud and assuming that k is equal to the theoretical Langevin rate coefficient in this case, as has been confirmed by the measurement of the state-dependent rate coefficients described in the main text.

If alternatively the experimental result for the effective rate coefficient of the O_2^+ + Rb reaction as quoted in the main text ($k_{\mathrm{eff}}(\mathrm{O}_2) = 2.9(8) \times 10-\ \mathrm{cm}^3\ \mathrm{s}^{-1}$) is used instead of the theoretical Langevin rate coefficient ($k_s^{\mathrm{L}}(\mathrm{O}_2) = 3.3 \times 10^{-9}\ \mathrm{cm}^3\ \mathrm{s}^{-1}$) to determine the Rb densities n_{avg}, one obtains the rate coefficients $k_s(\mathrm{N}_2) = 1.3(2) \times 10^{-9}\ \mathrm{cm}^3\ \mathrm{s}^{-1}$ and $k_p(\mathrm{N}_2) = 1.09(11) \times 10^{-8}\ \mathrm{cm}^3\ \mathrm{s}^{-1}$ for the reactions of N_2^+ with Rb in its $^2S_{1/2}$ and $^2P_{3/2}$ state, respectively. These results agree with the values quoted in the main text within their uncertainty limits.

In the experiments in which the transverse cooling lasers were switched on during transit (bright shuttling), the state populations and rate coefficients were obtained as above and k_p was calculated according to

$$k_p = \frac{(k - k_s) p_s}{p_p}, \tag{4}$$

with k_s determined from the dark shuttling experiments.

Supplementary Note 2 Classical capture model for Rb $((5p)\ ^2P_{3/2})$

Because Rb in its $(5p)\ ^2P_{3/2}$ state possesses a permanent quadrupole moment, a treatment of classical capture of the atom by an ion must also include the ion-permanent quadrupole interaction in addition to the Langevin (ion-induced dipole) interaction [3, 4]. The relevant long-range interaction potential reads

$$V(R) = \frac{C_3}{R^3} + \frac{C_4}{R^4} \tag{5}$$

with R the ion-atom distance and [5]

$$C_3 = (-1)^{l+\Lambda} \begin{pmatrix} l & 2 & l \\ -\Lambda & 0 & \Lambda \end{pmatrix} \langle l || \mathrm{Q}_2 || l \rangle, \tag{6}$$

$$C_4 = -\frac{1}{2}\left(\alpha_0 + \frac{3\Lambda^2 - 6}{6}\alpha_2\right). \tag{7}$$

Here, C_3 and C_4 denote the coefficients for the charge-quadrupole and the charge-induced dipole potential terms, respectively.

Based on Equation (5), the effective (centrifugally corrected) interaction potential V_{eff} is given by

$$V_{\mathrm{eff}}(R) = -\frac{C_4}{R^4} + \frac{C_3}{R^3} + \frac{E_{\mathrm{T}} b^2}{R^2}, \tag{8}$$

where E_{T} is the translational energy and b the impact parameter of the collision. The C_4 coefficient in (7) was calculated with the polarisabilities $\alpha_0 = 127\,\text{Å}^3$ and $\alpha_2 = -24.2\,\text{Å}^3$ from Ref. [6].

According to the treatment outlined in Refs. [4, 7, 8], the classical capture rate coefficient is calculated as

$$k = \pi b_{\mathrm{max}}^2 \sqrt{\frac{2E_{\mathrm{T}}}{\mu}}, \tag{9}$$

where μ is the reduced mass of the collision. The maximum impact parameter b_{max} in Equation (9) is calculated by first locating the maximum of the centrifugal barrier of the effective potential:

$$\left.\frac{\partial V_{\mathrm{eff}}}{\partial R}\right|_{R=R_{\mathrm{max}}} = \frac{4C_4}{R^5} - \frac{3C_3}{R^3} - \frac{2E_{\mathrm{T}} b^2}{R^3} = 0, \tag{10}$$

from which the position of the centrifugal barrier R_{max} is obtained according to:

$$R_{\mathrm{max}} = \frac{-3C_3 \pm \sqrt{(-3C_3)^2 + 32 E_{\mathrm{T}} b^2 C_4}}{4 E_{\mathrm{T}} b^2}. \tag{11}$$

From setting $V_{\mathrm{eff}}(R_{\mathrm{max}}) = E_{\mathrm{T}}$, one obtains an implicit equation for b_{max}

$$0 = E_{\mathrm{T}}(R_{\mathrm{max}}^4) - E_{\mathrm{T}} b_{\mathrm{max}}^2 (R_{\mathrm{max}}^2) - C_3 R_{\mathrm{max}} + C_4, \tag{12}$$

$$\tag{13}$$

from which b_{max} for Equation (9) is obtained.

Supplementary References

[1] Hall, F. H. J., Aymar, M., Bouloufa-Maafa, N., Dulieu, O. & Willitsch, S. Light-assisted ion-neutral reactive processes in the cold regime: Radiative molecule formation versus charge exchange. *Phys. Rev. Lett.* **107**, 243202 (2011).

[2] Hall, F. H. J. *et al.* Ion-neutral chemistry at ultralow energies: Dynamics of reactive collisions between laser-cooled Ca^+ ions and Rb atoms in an ion-atom hybrid trap. *Mol. Phys.* **111**, 2020-2032 (2013).

[3] Hall, F. & Willitsch, S. Millikelvin reactive collisions between sympathetically cooled molecular ions and laser-cooled atoms in an ion-atom hybrid trap. *Phys. Rev. Lett.* **109**, 233202 (2012).

[4] Zhang, D. & Willitsch, S. Cold chemistry: Molecular scattering and reactivity near absolute zero. In Dulieu, O. & Osterwalder, A. (eds.) *Cold Chemistry: Molecular Scattering and Reactivity Near Absolute Zero*, p. 496-536 (RSC Publishing, 2017).

[5] Krych, M. & Idziaszek, Z. Description of ion motion in a Paul trap immersed in a cold atomic gas. *Phys. Rev. A* **91**, 023430 (2015).

[6] Krenn, C., Scherf, W., Khait, O., Musso, M. & Windholz, L. Stark effect investigations of resonance lines of neutral potassium, rubidium, europium and gallium. *Z. Phys. D* **41**, 229–233 (1997).

[7] Gioumousis, G. & Stevenson, D. P. Reactions of Gaseous Molecule Ions with Gaseous Molecules. V. Theory. *J. Chem. Phys.* **29**, 294-299 (1958).

[8] Levine, R. D. *Molecular Reaction Dynamics* (Cambridge University Press, Cambridge, 2005).

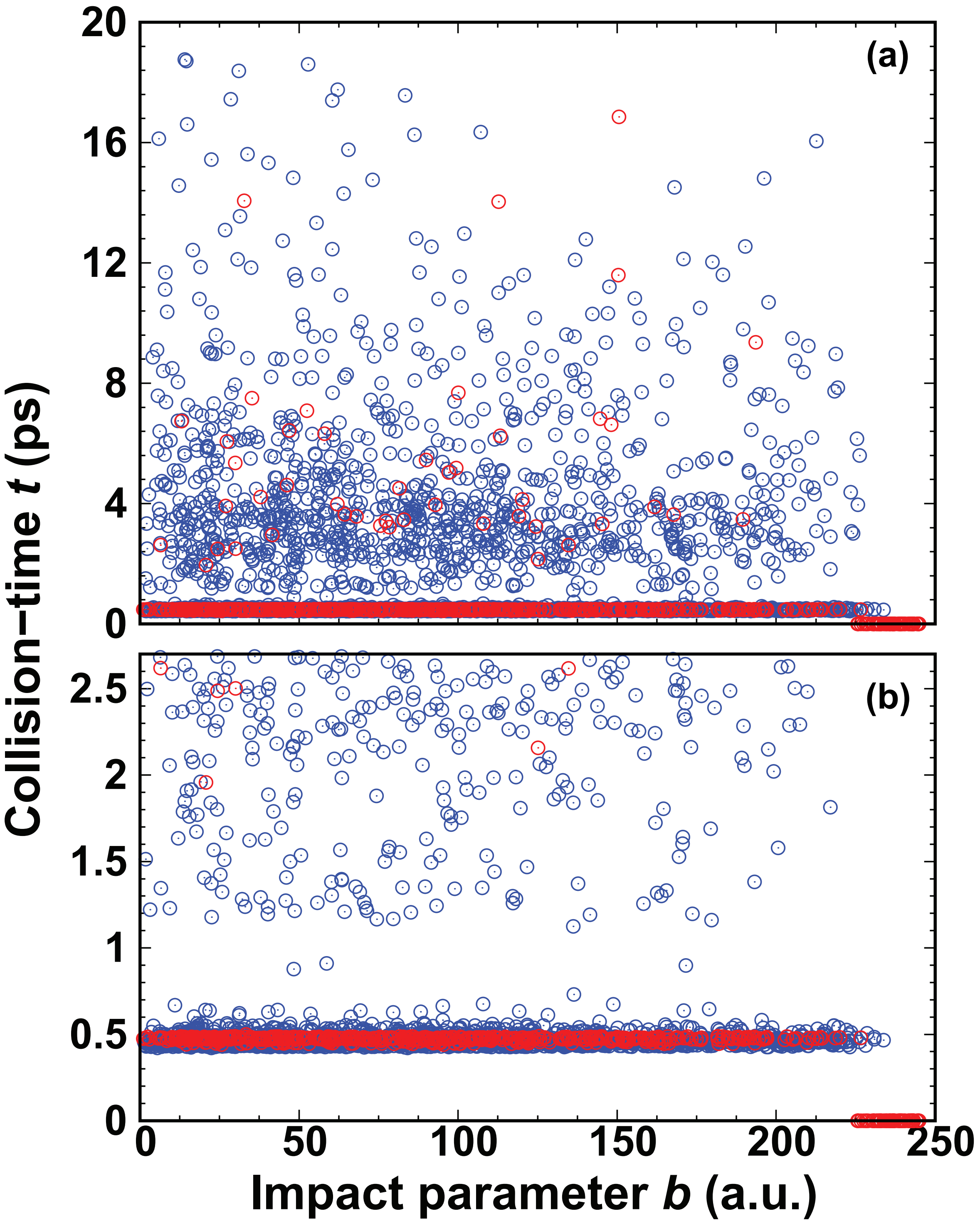

Supplementary Figure 1: **Collision times of charge-transfer reactions between N_2^+ and Rb.** (a) Collision-time as a function of impact parameter at 20 mK/k_B. Charge transfer trajectories are shown as blue circles and non-reactive trajectories are shown as red circles. (b) Magnified view of the region of low collision times in panel (a).

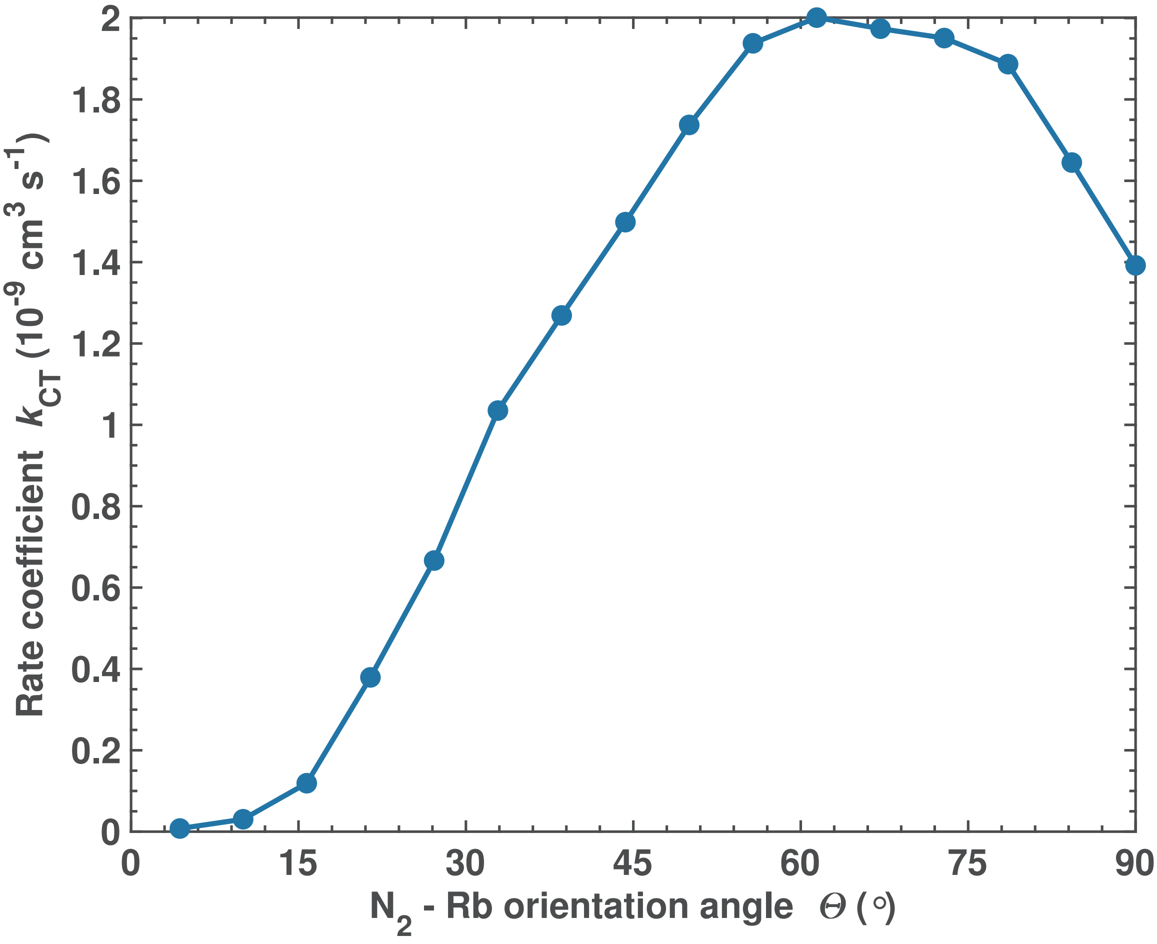

Supplementary Figure 2: **Orientation dependence of charge-transfer rate coefficients.** One-dimensional quantum rate coefficient for charge transfer between N_2^+ and Rb as a function of the N_2^+-Rb orientation angle θ.
